# Characteristics of familial pancreatic cancer families with additional colorectal carcinoma

**DOI:** 10.1007/s10689-023-00328-1

**Published:** 2023-01-31

**Authors:** Bettina Lehman, Elvira Matthäi, Norman Gercke, Ulrike W. Denzer, Jens Figiel, Timo Hess, Emily P. Slater, Detlef K. Bartsch

**Affiliations:** 1 Departments of Visceral, Thoracic and Vascular Surgery, Philipps University Marburg, Baldingerstrasse, 35043 Marburg, Germany; 2 Gastroenterology and Endocrinology, University Hospital Marburg, Marburg, Germany; 3 Centre for Human Genetics, University Hospital Marburg, Marburg, Germany

**Keywords:** Familial pancreatic cancer, Colorectal cancer, Mutations, Phenotype, Genotype

## Abstract

Familial pancreatic cancer (FPC) is a rare hereditary tumor entity with broad phenotypic heterogeneity, including colorectal carcinoma (CRC) in some families. The underlying factors for this co-occurrence are still not well evaluated. FPC families in the National Case Collection of Familial Pancreatic Cancer with an additional occurrence of CRC were analyzed regarding the phenotype, genotype and recommendation for a clinical screening program. The total cohort of 272 FPC families included 30 (11%) families with at least one CRC case. The proportion of affected family members with PDAC was 16.1% (73/451) compared to 9.3% of family members with CRC (42/451, p < 0.01). Females were affected with PDAC in 49% (36/73) and CRC in 38% (16/42). The median age of PDAC was 63 compared to 66 years in CRC, whereas 8 (26.6%) of families had an early onset of PDAC and 2 (6.7%) of CRC. Seventeen families had 2 or more affected generations with PDAC and 6 families with CRC. Eleven (9.6%) of affected patients had both PDAC and CRC. Potentially causative germline mutations (2 *ATM*, 1 *CDKN2a*, 1 *MLH1*, 1 *PALB2*) were detected in 5 of 18 (27.7%) analyzed cases. These findings provide a step forward to include the phenotypic and genotypic characteristics of FPC-CRC families for the genetic counseling and management of these families. Nevertheless, results need to be verified in a larger patient cohort beforehand.

## Introduction

Pancreatic ductal adenocarcinoma (PDAC) is one of the most aggressive and lethal tumor entities, with an average five-year survival rate of less than 9% [1]. This poor prognosis is mainly attributed to the late onset of subtle or nonspecific symptoms, in combination with limited treatment options at the time of diagnosis [1]. Despite recent breakthroughs for various types of cancer, early diagnosis and screening of PDAC remains challenging, due to the extensive heterogeneity of low-frequency genetic mutations [2]. Nevertheless, having two or more first-degree relatives with PDAC defined as so-called familial pancreatic cancer (FPC) syndrome [3, 4] is still one of the major risk factors (10% of incidences) for developing PDAC [3]. Therefore, several international tumor registries and collections were founded, including the North American National Familial Pancreatic Tumor Registry (NFPTR), the German National Case Collection of Familial Pancreatic Cancer and the European Registry of Hereditary Pancreatitis and Familial Pancreatic Cancer (EUROPAC) [5–7]. The common goal is to identify signature gene clusters and related phenotypes in affected families for an improvement in genetic counseling and earlier detection in a prospective PDAC screening program [8]. Recent studies failed to identify general driver mutations for FPC susceptibility, while even less is known about the phenotypic variants in FPC families [2, 9]. Nevertheless, registered families can be roughly divided into groups of “pure” FPC and those with additional co-occurrence of other tumours, such as breast, colon, lung, or prostate cancer [5, 10]. Within this area of study, we recently detected some predisposing low penetrance genes that may relate to an additional susceptibility to breast cancer in some cases of the FPC families [5]. The current study aimed to further fill the knowledge gaps of undefined phenotypic and genotypic factors as well as support future studies for prospective PDAC screening in FPC associated with colorectal cancer (FPC-CRC).

### Patients and methods

The German National Case Collection of Familial Pancreatic Cancer (FaPaCa) is a tumor collection funded by the Deutsche Krebshilfe in 1999 [5]. It was established to investigate the phenotype and genotype of families with two or more first-degree relatives with PDAC, referred to as FPC families. Moreover, the FaPaCa collection offers a screening program for these FPC family relatives. Initially, the screening age started at 40, until it was set back to 50 years in 2016. Alternatively, the screening of family members can begin at the age corresponding to 10 years before the earliest recorded onset of PDAC within the family history. An exclusion criteria for the screening program is the documented evidence of another inherited tumor syndrome. An FPC family member carrying a predisposing mutation, e.g. BRCA2, will be classified as an individual at risk (IAR), if at least one relative was affected by a PDAC. This screening program entails an annual physical examination, collection of blood samples, determination of serum HbA1c, amylase, GOT, GPT, bilirubin, and CA19-9, and imaging with MRI plus magnetic resonance cholangiopancreatography (MRCP) and endosonography [11,8]. The FPC family members were referred by their physicians, or by contacting the FaPaCa study office based on contact information on the FaPaCa website starting July 1999 (http://www.fapaca.de). As part of the registration process, all eligible families were genetically counseled, and a three-generation family pedigree was constructed.

The current report analyzed the genotype and phenotype of FPC families with an additional occurrence of CRC. The age of diagnosis of PDAC and CRC was retrieved from the three-generation pedigrees and early age of onset was defined as the occurrence of PDAC or CRC prior to the age of 50 years in a family [8, 12–14]. All PDAC and CRC diagnoses were confirmed by review of medical records and death certificates. Furthermore, the pathology report defined the pancreatic lesions of operated patients PDAC, pancreatic intraepithelial neoplasia type (PanIN) 1–3 or intraductal papillary mucinous neoplasia (IPMN) with low or high-grade dysplasia.

All patients with PDAC and/or FPC-CRC families with available blood samples, given their informed consent was collected, were subjected to mutation analysis by multi-cancer gene panel, including cancer predisposition genes [9, 15–20]. In addition, resected PDACs of affected patients from FPC-CRC families also underwent immunohistochemical analysis of mismatch repair genes *MLH1*, *MSH2*, *PMS2* and *PMS6*. If loss of expression was detected in one of these genes, Sanger sequencing of the respective gene was performed in the corresponding germline. If a deleterious germline mutation was identified in the tested patient, genetic counseling and a predictive genetic test of this mutation were offered to all family members. The results were explained to the family members in a follow-up genetic counseling. The FaPaCa collection and screening program, as described here, was approved by the Ethics Committee of the Philipps-University of Marburg (36/1997, last amendment 9/2010), while all participating family members provided their written informed consent.

### Statistics

All descriptive information from the FaPaCa family members was compiled. These included age, gender, number of family members with PDAC, earliest age of onset in the family and known germline mutations. The early-onset was defined as the diagnosis of PDAC as well as CRC prior to the age of 50 years [8, 12–14].

The chi-square, Fisher’s exact test, t-test and Wilcoxon rank sum test were completed for categorical and numerical variables, to compare the characteristics of patients. Two-tailed p values < 0.05 were considered to be statistically significant. Analyses were performed with Prism GraphPad Software, Inc.

## Results

Based on the FaPaCa collection of 272 FPC families, we identified 30 (11%) families with at least one CRC case in their family history. During follow-up screens, only one of these 30 families emerged to fulfill the criteria of Hereditary nonpolyposis colorectal cancer (HNPCC) [21]. Of the 451 registered family members of these FPC-CRC 30 families, 73 (16.1%) had PDAC and 42 (9.3%) had developed CRC.

**Fig. 1:**
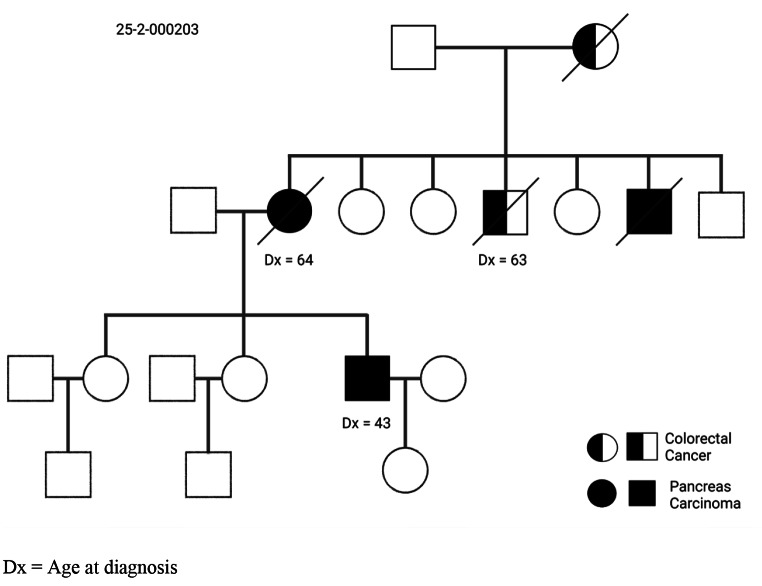
Pedigree of a representative FPC-CRC

We further investigated the distinct phenotypes of the two groups. The proportion of affected female patients with 49% (36/73) with PDAC in FPC families was similar to the general population (48% females) [22]. Furthermore, CRC occurrence in females of FPC families was 38% (16/42, p < 0.01), compared to CRC occurrence in the general population with 41% females [23, 24]. However, the median age at the time of diagnosis was lower in PDAC at 63 years (37–83), while CRC demonstrated a median age of 66 (44–90) years (p < 0.01). Furthermore, 8 (26.6%) families had an early-onset of PDAC < 50 years compared to only 2 families (6.7%) with an early onset of CRC < 50 years. Seventeen (57%) families had two or more affected generations with PDAC and 6 (20%) families with CRC (p > 0.05). Eleven of 115 (9.6%) affected patients had both PDAC and CRC. The characteristics of FPC-CRC families are summarized in Table 1. A representative FPC-CRC family is shown in Fig. 1.

**Table 1:** Characteristics of FPC-CRC families

Characteristic	FPC/CRC families (n = 30)
Total number family members	451
Total number PDAC cases	73
FDR with PDAC/family 2 3 > 3	20 9 0
1 affected generation with PDAC	13
2 affected generations with PDAC	17
3 affected generation with PDAC	0
median age at Dx PDAC	63 (37–83)
No. families with early age of onset of PDAC (< 50 yrs.)	8
females with PDAC	36/73 (49%)
total number of CRC cases	42
FDR with CRC/family 1 2 3 or more	21 7 2
1 affected generation with CRC	24
2 affected generations with CRC	5
3 affected generations with CRC	1
median age at Dx CRC	66 (44–90)
No. families with early age of onset of CRC (< 50 yrs.)	2
females with CRC	16/42 (38%)
No. families fulfilling criteria of HNPCC	1
Pats. with both PDAC and CRC	11 (9.6%)
No. of families with other tumor types	20 (66.7%)
Cancer types	breast cancer (7); melanoma (4); lung cancer (4); renal/bladder cancer (3); endometrium cancer (3); prostate cancer (2); esophageal (2); gastric cancer (1); ovarian cancer (1); hepatocellular carcinoma (1); medulloblastoma (1); basiloma (1), thyroid carcinoma (1), head/neck carcinoma (1)
Families with deleterious germline mutations	5 of 18 (27.8%)
Mutated genes (4)	MLH1(1), PALB2(1), CDKN2A(1), ATM(2)

Mutated genes: MLH1: c.1835_1837delTTG; p.(Val612del), PALB2: c.509_510del; p.(Arg170Ilefs14)

CDKN2A: c.301G > T; p.(Gly101Trp), ATM: c.381del; p.(Val128) and c.7630-2 A > C; p.(Leu2544_Glu2596del)

No.: Number of

Pats.: Patients

Dx: Age at diagnosis

FDR: First degree relative

In the next step, we performed mutation analysis of 18 FPC-CRC family members, where patient consent and tissues were readily available, including 4 individuals with co-occurrence of FPC and CRC. Here, we identified potentially causative germline mutations (2 *ATM*, 1 *CDKN2a*, 1 *MLH1*, 1 *PALB2*) in 5 (27%) individuals (Table 2). This mutation spectrum is distinct from other previously described gene mutations in FPC (*BRCA1, BRCA2, CHEK2, MSH2, MSH6*), which remained undetected in the analyzed 18 FPC-CRC family members.

**Table 2:** Altered genes in FPC-CRC families

Gene analyzed	FPC-CRC families tested	FPC-CRC families with alterations (n = 5)	Effect of Alteration: Deleterious/Unclassified/Benign
**BRCA1**	18	0	n.a.
**BRCA2**	18	0	n.a.
**PALB2**	18	1	pathogenic
**CDKN2a**	18	1	likely pathogenic
**ATM**	18	2	likely pathogenic
**CHEK2**	18	0	n.a.
**MLH1**	18	1	pathogenic
**MSH2**	18	0	n.a.
**MSH6**	18	0	n.a.
**PMS2**	18	0	n.a.

n.a.: not applicable

From these 30 FPC-CRC families, 25 Individuals at risk (IAR) with a median age of 49 (36–62) years participated in a prospective annual PDAC screening program with a median of 2 (1–18) screening visits. Upon imaging, small (< 10 mm) cystic lesions were detected in 20 (80%) of IAR and 2 (8%) IARs underwent surgery because of suspicious lesions upon imaging in the pancreatic head. A 56 year old male underwent pylorus-preserving pancreaticoduodenectomy (PPPD) and pathology revealed pancreatic intraductal papillary mucinous neoplasms (BD-IPMN) with multifocal PanIN2 lesions. This IAR was not a carrier of the predisposing PDAC-germline mutation. Another 83 year old male carrying a *MLH1* mutation had total pancreatectomy for a solid lesion. Pathology reported a PDAC UICC stage 3 (pT3, N1, M0).

## Discussion

There is still an overwhelming knowledge gap in the clinical management of FPC families, since only in about 10–15% of FPC cases predisposing germline mutations have been detected so far [5, 25]. FPC can be roughly divided into two groups, namely pure FPC families (~ 40% of families) and those associated with co-occurrences of other tumor types, most frequently breast cancer (31%), CRC (11%), or melanoma (9.7%) [5, 26]. In this study we sought to take advantage of the FaPaCa collection [5] to analyze, for the first time, the phenotype and genotype of FPC families associated with additional CRC.

A total of 9.3% (42/451) FPC family members developed CRC, compared to the lower incidence of CRC in the general German population with 0.08% (84.8/100,000) as reported in 2019 [24, 27].

Our analysis demonstrated that almost half of the patients in the FPC-CRC families with PDAC were female 49% (36/73), whereas only 38% (16/42) females demonstrated CRC (Table 1). CRC, in general, is a disease strongly influenced by gender and biased towards males [28]. Underlying reasons include behavioral CRC risk factors, e.g., increased consumption of red, processed meat, alcohol, and smoking, in addition to a greater likelihood to deposit visceral fat [29–31]. These factors can potentially also play a role in the gender bias in FPC-CRC families.

In this study, the median age of diagnosis was relatively low in FPC-CRC families with 63 years for PDAC and 66 years for CRC, respectively (Table 1). Nevertheless, there was no difference in the previously reported median age of PDAC onset in FPC families with 63 (35–91) years [5] compared to the presented FPC-CRC families with 63 (37–83) years (Table 1). Overall, there were 9.3% (42/451) of FPC family members with CRC in this FPC-CRC cohort. Recently, there was more effort put into context-dependent driver gene discovery, leading to either exclusive or co-occurrence of cancer entities in one patient [32]. Therefore, we further investigated the genetic makeup of FPC-CRC families to analyze the relationship of cross-cancer mutation patterns, where a co-occurrence in both FPC and CRC may indicate synergistic impact on tumorigenesis This was also an opportunity to expand the panel of genes under study and discover new candidate germline genes to be further validated prospectively.

Here, we detected four potentially causative germline mutated genes (2 *ATM*, 1 *CDKN2a*, 1 *MLH1*, and 1 *PALB2*) in 5 of 18 (27.7%) FPC-CRC families (Table 2). This is a relatively high frequency of gene mutations, compared to the rate of detected potentially deleterious germline mutations in reported pure FPC families about 10% [5, 9, 33–37]. The *ATM*, *CDKN2A*, *MLH1*, and *PALB2* mutations in FPC-CRC families have separately been associated with a high to moderate risk of CRC [38]. *ATM* and *MLH1* are classified as high penetrance modifiers in CRC [39], while *CDKN2A* and *PALB2* have been reported as moderate-risk CRC susceptibility genes [40]. In brief, ATM is a key player in the maintenance of genomic integrity during DNA repair. Previous studies identified a connection between *ATM* mutations and an increased predisposition in solid tumors, including pancreas, breast, gastric, lymphoid, CNS, skin, and others [41]. Furthermore, *ATM* mutations may result in resistance to chemotherapeutic therapies. To date, its potential role as a predictive and prognostic biomarker has not been fully investigated. Further pathogenic gene mutations of *MLH1* and *CDKN2A* have been recently described in pancreatic and upper gastrointestinal tract tumors but have not been evaluated in the setting of familial FPC-CRC predisposition [42]. Whereas *BRCA2* mutations, have been reported to play a role in neoplasia in hereditary breast and ovarian cancer (HBOC) and may be involved in up to half of hereditary breast cancer [43]. Instead of *BRCA2* itself, this study detected mutations in FPC-CRC kindreds in *PALB2*, a co-localizer and partner gene to *BRCA2*, which is also proposed to be involved in FPC [44]. Consequently, *ATM*, *CDKN2A*, *MLH1*, and *PALB2* mutations in FPC-CRC families might biologically and clinically relevant for both PDAC and CRC development. This should be further investigated in a larger cohort of familial FPC-CRC patients. Since the general issue in the field is the relatively small number of families and samples, the establishment of multi-center cohorts would be beneficial to increase the cohorts for statistical power and relevance for clinical practice.

Despite the small patient cohort, the assumption that the co-occurrence between FPC and CRC is merely a coincidence is relatively unlikely, since 11 (15%) of 73 patients with PDAC also had CRC, but only 1 of these patients belonged to a hereditary non-polyposis colorectal cancer (HNPCC) family. In comparison, HNPCC is one of the most common familial aggregations of hereditary cancer in the gastrointestinal tract [45], but its association with PDAC was reported between 1.3 and 6%, and is not as striking [46–48]. Similar low co-occurrences are seen in Familial Adenomatous Polyposis [21], which is related to the germline mutations of the APC gene [49]. Initial studies from 1993 suggested that FAP patients have a 4.5 times increased risk of developing PDAC compared to the general population [50]. However, a more recent study from the Johns Hopkins Polyposis registry found only 4 (0.3%) patients with PDAC in their cohort of 1391 patients with FAP [50, 51], while Driffa et al. did not report any cases of PDAC in their cohort of 127 FAP patients between 1990 and 2009 [52]. Therefore, the FPC-CRC cases are relatively frequent in comparison to FPC-HNPCC or FPC-FAP cases, and potentially more than just an arbitrary coincidence.

Recent expert guidelines recommend a PDAC screening in IAR of FPC families [33], especially in mutation carriers of predisposing genes. During screening, cystic lesions are frequently detected in 40 to 60% of IAR [5, 21, 33, 53, 54]. Twenty-five IAR from our 30 FPC-CRC families participated in a board approved controlled screening program and 20 (80%) showed cystic lesions (Table 3), which are somehow pathognomonic for FPC [55]. The diagnostic yield of screening, defined as the detection of high grade pancreatic intraepithelial neoplasia (PanIN) or PDAC lesions, is reported overall between 1.5 and 8% [5, 33, 53, 56] and up to 9.3% in carriers of BRCA1/2, CDKN2a and ATM mutation carries [53]. In the present study, 2 of 25 (8%) IAR under screening had histopathological proven relevant pancreatic lesions (Table 3), which is a comparably high rate compared to mutation carriers of predisposing genes [10, 26]. This could be a characteristic of FPC-CRC families, which might be considered during counselling and for inclusion of these IAR into PDAC screening programs.

We observed interesting phenotypic and genotypic findings for FPC-CRC families, but a major limitation of our study was the relatively small number of families, as well as only partial availability of all patient material for mutational sequencing analysis. The same issue holds true for the limited number of IARs enrolled in the screening program. Therefore, our observations should be interpreted with caution and the results need to be verified by a larger number of FPC-CRC families to determine, whether these reported characteristics can be confirmed and can be translated into the management of these families.

**Table 3:** Characteristics of IAR from FPC-CRC families in PDAC screening

	FPC-CRC families
Families in Screening	17
IARs in screening	25
Gender f/m	13/12
IAR with predisposing germline mutations for PDAC	1 (4%)
median follow-up, month (range)	47 (1-219)
median screening visits (range)	2 (1–18)
median age of IAR at first screening, years	49 (36–62)
IAR with cystic lesions on imaging	20 (80%)
IAR with solid lesions on imaging	1
IAR who underwent surgery	2 (8%)
total pancreatectomy	1
PPPD	1
Histology	
PDAC	1 (pT3N1M0)
Multifocal PanIN2 and BD-IPMN with low grade dysplasia	1

IAR- individual at risk

PPPD- pylorus preserving pancreaticoduodenectomy

BD-IPMN – Branch-duct pancreatic intraductal papillary mucinous neoplasm
